# Comparison of Provisional with Final Notifiable Disease Case Counts — National Notifiable Diseases Surveillance System, 2009

**Published:** 2013-09-13

**Authors:** Nelson Adekoya, Henry Roberts

**Affiliations:** Div of Notifiable Diseases and Healthcare Information, Public Health Surveillance and Informatics Program Office; Div of Viral Hepatitis, National Center for HIV/AIDS, Viral Hepatitis, STD, and TB Prevention, CDC

States report notifiable disease cases to CDC through the National Notifiable Diseases Surveillance System (NNDSS). This allows CDC to assist with public health action and monitor infectious diseases across jurisdictional boundaries nationwide. The *Morbidity and Mortality Weekly Report* (*MMWR*) is used to disseminate these data on infectious disease incidence. The extent to which the weekly notifiable conditions are overreported or underreported can affect public health understanding of changes in the burden, distribution, and trends in disease, which is essential for control of communicable diseases (1). NNDSS encourages state health departments to notify CDC of a case when initially reported. These cases are included in the weekly provisional counts. The status of reported cases can change after further investigation by the states, resulting in differences between provisional and final counts. Increased knowledge of these differences can help in guiding the use of information from NNDSS. To quantify the extent to which final counts differ from provisional counts of notifiable infectious disease in the United States, CDC analyzed 2009 NNDSS data for 67 conditions. The results of this analysis demonstrate that for five conditions, final case counts were lower than provisional counts, but for 59 conditions, final counts were higher than provisional counts. The median difference between final and provisional counts was 16.7%; differences were ≤20% for 39 diseases but >50% for 12. These differences occur for various diseases and in all states. Provisional case counts should be interpreted with caution and an understanding of the reporting process.

Reporting of cases of certain diseases is mandated at the state or local level, and states, the Council of State and Territorial Epidemiologists (CSTE), and CDC establish policies and procedures for submitting data from these jurisdictions to NNDSS. Not all notifiable diseases are reportable at the state level, and although disease reporting is mandated by legislation or regulation, state reporting to CDC is voluntary. States send reports of cases of nationally notifiable diseases to CDC on a weekly basis in one of several standard formats. Amended reports can be sent, as well as new reports. Cases are reported by week of notification to CDC. Cases reported each week to CDC and published in *MMWR* are deemed provisional. The NNDSS database is open throughout the year, allowing states to update their records as new information becomes available. Annually, CDC provides each state epidemiologist with a cutoff date (usually 6 months after the end of the reporting year) by which all records must be reconciled and no additional updates are accepted for that reporting period. After the database is closed, final case counts, prepared after the states have reconciled the year-to-date data with local reporting units, are approved by state epidemiologists as accurate reflections of final case counts for the year and are published in the *MMWR Summary of Notifiable Diseases — United States.* Data for 2009 were published in 2011 (2).

CDC’s publication schedule allows states time to complete case investigation tasks. To examine the extent that provisional counts of infectious diseases differ from final counts, CDC compared the cumulative case counts published for week 52 of 2009 in the *MMWR* of January 8, 2010 to the case counts published in the NNDSS final data set for 2009 (cutoff date of June 2010) published in *MMWR* on August 20, 2010. To assess whether discrepancies between provisional and final counts were more common in specific states or regions, or everywhere, reporting was examined, by state, of four diverse diseases: one sexually transmitted disease (*Chlamydia trachomatis*, genital infection), one vaccine-preventable disease (pertussis), one foodborne disease (salmonellosis), and one vectorborne disease (Lyme disease). Data are not presented for tuberculosis and human immunodeficiency virus (HIV)/acquired immunodeficiency syndrome because these data are published quarterly rather than weekly in *MMWR*. Weekly reports of these conditions to the public health community are of limited value because of differences in reporting patterns for these diseases, and long-term variations in the number of cases are more important to public health practitioners than weekly variations (3).

Reported data for 67 notifiable diseases were reviewed. Final counts were lower than provisional counts for five diseases, the same as provisional counts for three, and higher for 59 (Table 1). The median difference between final and provisional counts was 16.7%; differences were ≤20% for 39 diseases but >50% for 12. Among diseases with ≥10 cases reported in 2009, final counts were lower than provisional counts for just four: invasive *Haemophilus influenzae* disease, ages <5 years, unknown serotype (final: 166, provisional: 218); acute hepatitis C (final: 782, provisional: 844); toxic-shock syndrome, other than streptococcal (final: 74, provisional: 76); and influenza-associated pediatric mortality (final: 358, provisional: 360). Final counts were higher than provisional counts for 51 diseases. The greatest percentage differences between provisional and final case counts were for arboviral disease, West Nile virus (neuro/nonneuro) (final: 720, provisional: 0); mumps (final: 1,991, provisional: 982); and Hansen disease (final: 103, provisional: 59).

Examining four diverse but commonly reported diseases in detail revealed no consistent association between state or region and the magnitude of the discrepancy between final and provisional counts (Table 2). For *Chlamydia trachomatis*, genital infections, the final case count was 13.1% higher than the provisional count nationally; it was <2% lower everywhere and ≥20% higher in six states. Two states, Arkansas and North Carolina, reported no cases provisionally, but reported final case counts of 14,354 and 41,045, respectively. For Lyme disease, the final case count was 29.2% higher than the provisional count nationally. Only 23 jurisdictions reported >100 cases, including 21 states, upstate New York, and New York City. Of these, four states reported a final count lower than their provisional count (range: 13.4%–29.2%) and eight jurisdictions reported final counts ≥20% higher. The greatest percentage differences between provisional and final case counts were in Connecticut (final: 4,156, provisional: none), Minnesota, (final: 1,543, provisional: 169), Texas (final: 276, provisional: 48), and New York City (final: 1,051, provisional: 262). For pertussis, the final case count was 24.8% higher than the provisional count nationally; it was <2% lower everywhere and ≥20% higher in 18 states and the District of Columbia (DC). Of the five states that reported >1,000 cases, the states with the greatest percentage differences between provisional and final counts were Minnesota (final: 1,121, provisional: 165) and Texas (final: 3,358, provisional: 2,437). For salmonellosis, the final case count was 10.6% higher than provisional count nationally. Six states reported a final count lower than their provisional count (range: 0.1%–2.9%) and nine states plus DC reported final counts ≥20% higher, the highest being DC (final: 100, provisional: 26), Louisiana (final: 1,180, provisional: 599), and Indiana (final: 629, provisional: 349).

## Editorial Note

The findings in this report corroborate previous observations that provisional NNDSS data should be interpreted with caution (1,4,5). The primary appeal of provisional counts is timeliness; in comparison, final counts are more complete and accurate. As additional information is collected during investigations, final case counts might be higher or lower than the provisional counts. Local and state health departments collect reportable surveillance data primarily to assist with disease control and prevention efforts (i.e., to monitor local outbreaks of infectious diseases), to measure disease burden among high-risk populations, and to assess effectiveness of local interventions. At the national level, these data can be compared with baseline data to detect unusual disease occurrences. Final data sets are useful in monitoring national trends and for determining the effectiveness of national intervention efforts. In 2009, final case counts did not differ from end-of-year provisional counts by >20% for two thirds of the 67 notifiable diseases examined. Understanding how provisional counts relate to final counts is essential for interpreting provisional data (6,7).

What is already known on this topic?

Provisional counts of notifiable diseases usually differ from final counts; they are most often lower.

What is added by this report?

In 2009, finalized case counts were higher than the provisional case counts for 59 of 67 notifiable diseases. The median difference between final and provisional counts was 16.7%; differences were ≤20% for 39 diseases but >50% for 12. These differences occur, to a greater or lesser extent, for a wide variety of diseases and in all states.

What are the implications for public health practice?

Notifiable disease data are subject to case reclassification leading to undernotification or overnotification. Provisional case counts should be interpreted with caution because of the reporting process. The primary appeal of provisional counts is timeliness; in comparison, final counts are more complete and accurate.

Final counts might be higher than provisional counts for several possible reasons: 1) as amended records are sent by states during the notification process, cases might be reclassified among confirmed, probable, suspected, and not-a-case categories; 2) states vary in their practices regarding when they report cases with incomplete data or that are under investigation, leading to variable delays; 3) allocation of cases to a state can be delayed; 4) laboratory testing, case investigation, and data entry can be delayed as a result of temporary staff absences (e.g., leave, furlough, or turnover); 5) states sometimes delay sending some reports to CDC until the end of the year; and 6) internal CDC data processing problems can cause a discrepancy.

The findings in this report are subject to at least one limitation. It was impossible to determine when final counts were known to the state and local jurisdictions so that they could take public health action. This report focuses only on counts published in *MMWR*. The jurisdictions might have been aware of final case counts sooner, and only notification to CDC was delayed. Although this study examined 1 year of data, previous research using multiple years of data for hepatitis A and B concluded that provisional data generally tend to underrepresent the final data counts for those conditions (1). The addition of more years to the current research, which examined multiple notifiable conditions and documents substantial differences across states, regions, and numerous conditions, would not be expected to change the overall results.

Interpreting weekly incidence data is complex because of surveillance system limitations. Nonetheless, health practitioners have to respond to public health threats based on preliminary surveillance information. In 2006, CDC and CSTE reconsidered data presentation formats and included additional information (e.g., 5-year weekly average, previous 52 weeks median, and maximum number of cases) to aid interpreting these data (3). However, the findings in this report illustrate that major challenges still exist in presenting and interpreting provisional data and highlights the need to examine specific factors that can contribute to late reporting of cases (e.g., late case reporting by providers to health departments or late reporting of cases by health departments to CDC) (4). Although information technology has improved notifiable disease reporting (8), NNDSS data remain subject to reporting artifacts. Understanding specific reasons for the variation between the provisional and final case counts for each condition can improve the use of provisional data for disease surveillance and notification.

## Figures and Tables

**TABLE 1:** Comparison of provisional and finalized notifiable diseases data — National Notifiable Diseases Surveillance System, 2009

Disease	Final data	Provisional data	Absolute change	Change (%)
Anthrax	1	—	1	
Arboviral disease, California serogroup (neuro/nonneuro)	55	41	14	(34.1)
Arboviral disease, Eastern equine (neuro/nonneuro)	4	4	0	(0.0)
Arboviral disease, Powassan (neuro)	6	1	5	(500.0)
Arboviral disease, St. Louis encephalitis (neuro/nonneuro)	12	10	2	(20.0)
Arboviral disease, West Nile virus (neuro/nonneuro)	720	—	720	
Botulism, total	118	92	26	(28.3)
Brucellosis	115	100	15	(15.0)
Chancroid	28	25	3	(12.0)
*Chlamydia trachomatis*, genital infections	1,244,180	1,100,230	143,950	(13.1)
Cholera	10	8	2	(25.0)
Coccidioidomycosis	12,926	12,729	197	(1.5)
Cryptosporidiosis, total	7,654	6,652	1,002	(15.1)
Cyclosporiasis	141	123	18	(14.6)
Ehrlichiosis, *Ehrlichia chaffeen*	944	801	143	(17.9)
Ehrlichiosis, *Ehrlichia ewingii*	7	6	1	(16.6)
Ehrlichiosis, *Anaplasma phagocytophilum*	1,161	690	471	(68.3)
Ehrlichiosis, undetermined	155	122	33	(27.0)
Giardiasis	19,399	17,548	1,851	(10.6)
Gonorrhea	301,174	260,530	40,644	(15.6)
*Haemophilus influenzae*, invasive disease, all ages, both sexes	3,022	2,896	126	(4.4)
*Haemophilus influenzae*, invasive disease, ages <5 yrs, serotype b	38	25	13	(52.0)
*Haemophilus influenzae*, invasive disease, ages <5 yrs, nonserotype b	245	203	42	(20.7)
*Haemophilus influenzae*, invasive disease, ages <5 yrs, unknown serotype	166	218	−52	(−23.9)
Hansen disease	103	59	44	(74.6)
Hantavirus pulmonary syndrome	20	12	8	(66.7)
Hemolytic uremic syndrome postdiarrheal	242	210	32	(15.2)
Hepatitis A, viral, acute	1,987	1,849	138	(7.5)
Hepatitis B, viral, acute	3,405	3,020	385	(12.7)
Hepatitis C, viral, acute	782	844	−62	(−7.4)
Influenza-associated pediatric mortality	358	360	−2	(−0.6)
Legionellosis	3,522	3,145	377	(12.0)
Listeriosis	851	755	96	(12.7)
Lyme disease, total	38,468	29,780	8,688	(29.2)
Malaria	1,451	1,169	282	(24.1)
Measles, total	71	61	10	(16.4)
Meningococcal disease, all serogroups	980	887	93	(10.5)
Mumps	1,991	982	1,009	(102.8)
Pertussis	16,858	13,506	3,352	(24.8)
Plague	8	7	1	(14.3)
Polio	1	—	1	
Psittacosis	9	9	0	(0.0)
Q fever, total	113	95	18	(19.0)
Rabies, animal	5,343	3,581	1,762	(49.2)
Rabies, human	4	4	0	(0.0)
Rocky Mountain spotted fever, total	1,815	1,393	422	(30.3)
Rubella	3	4	−1	(−25.0)
Rubella, congenital syndrome	2	1	1	(100.0)
Salmonellosis	49,192	44,468	4,724	(10.6)
Shiga toxin-producing *Escherichia coli* (STEC)	4,643	4,323	320	(7.4)
Shigellosis	15,931	14,581	1,350	(9.3)
Streptococcal disease, invasive group A	5,279	4,861	418	(8.6)
Streptococcal toxic-shock syndrome	161	125	36	(28.8)
*Streptococcus pneumoniae*, invasive disease, drug resistant, all ages	3,370	2,823	547	(19.4)
*Streptococcus pneumoniae*, invasive disease, drug resistant, ages <5 yrs	583	464	119	(25.7)
*Streptococcus pneumoniae*, invasive disease, nondrug resistant, ages <5 yrs	1,988	1,768	220	(12.4)
Syphilis, congenital	427	257	170	(66.2)
Syphilis, primary and secondary	13,997	12,833	1,164	(9.1)
Tetanus	18	14	4	(28.6)
Toxic-shock syndrome (other than streptococcal)	74	76	−2	(−2.6)
Trichinellosis	13	12	1	(8.3)
Tularemia	93	79	14	(17.7)
Typhoid fever	397	324	73	(22.5)
Vancomycin-intermediate *Staphylococcus aureus* (VISA)	78	70	8	(11.4)
Vancomycin-resistant *Staphylococcus aureus* (VRSA)	1	—	1	
Varicella (chickenpox morbidity)	20,480	16,944	3,536	(20.9)
Vibriosis	789	593	196	(33.1)

**TABLE 2:** Comparison of provisional and final reported cases of notifiable diseases for selected conditions, by state and area — National Notifiable Diseases Surveillance System, United States, 2009

	Chlamydia			Lyme disease			Pertussis			Salmonellosis		
				
Area	Final	Provisional	Change (%)	Final	Provisional	Change (%)	Final	Provisional	Change (%)	Final	Provisional	Change (%)
**United States**	**1,244,180**	**1,100,230**	**(13.1)**	**38,468**	**29,780**	**(29.2)**	**16,858**	**13,506**	**(24.8)**	**49,191**	**44,468**	**(10.6)**
**New England**	40,776	39,850	(2.3)	12,440	6,314	(97.0)	626	592	(5.7)	2,174	2,110	(3.0)
Connecticut	12,127	11,532	(5.2)	4,156	—		56	48	(16.7)	430	406	(5.9)
Maine	2,431	2,386	(1.9)	970	894	(8.5)	80	78	(2.6)	121	119	(1.7)
Massachusetts	19,315	19,538	(−1.2)	5,256	3,662	(43.5)	358	348	(2.9)	1,155	1,159	(−0.4)
New Hampshire	2,102	1,633	(28.7)	1,415	1,156	(22.4)	76	76	(0.0)	261	243	(7.4)
Rhode Island	3,615	3,614	(0.0)	235	212	(10.9)	45	31	(45.2)	144	122	(18.0)
Vermont	1,186	1,147	(3.4)	408	390	(4.6)	11	11	(0.0)	63	61	(3.3)
**Mid-Atlantic**	159,111	154,989	(2.7)	16,346	16,691	(−2.1)	1,222	1,101	(11.0)	5,514	5,001	(10.3)
New Jersey	23,974	21,181	(13.2)	4,973	4,163	(19.5)	244	158	(54.4)	1,132	802	(41.2)
New York (Upstate)	33,722	32,099	(5.1)	4,600	4,179	(10.1)	265	252	(5.2)	1,370	1,321	(3.7)
New York City	58,347	59,370	(−1.7)	1,051	262	(301.2)	98	92	(6.5)	1,253	1,171	(7.0)
Pennsylvania	43,068	42,339	(1.7)	5,722	8,087	(−29.2)	615	599	(2.7)	1,759	1,707	(3.1)
**Eastern North Central**	197,133	167,016	(18.0)	2,969	2,359	(25.9)	3,206	2,990	(7.2)	5,169	4,597	(12.4)
Illinois	60,542	48,929	(23.7)	136	126	(7.9)	648	570	(13.7)	1,484	1,294	(14.7)
Indiana	21,732	21,111	(2.9)	83	62	(33.9)	392	338	(16.0)	629	349	(80.2)
Michigan	45,714	44,873	(1.9)	103	99	(4.0)	900	854	(5.4)	960	911	(5.4)
Ohio	48,239	34,036	(41.7)	58	56	(3.6)	1,096	1,096	(0.0)	1,407	1,407	(0.0)
Wisconsin	20,906	18,067	(15.7)	2,589	2,016	(28.4)	170	132	(28.8)	689	636	(8.3)
**Western North Central**	70,396	66,205	(6.3)	1,693	303	(458.8)	2,840	1,678	(69.3)	2,679	2,472	(8.4)
Iowa	9,406	9,311	(1.0)	108	96	(12.5)	235	192	(22.4)	408	398	(2.5)
Kansas	10,510	9,798	(7.3)	18	14	(28.6)	240	146	(64.4)	398	269	(48.0)
Minnesota	14,197	12,222	(16.2)	1,543	169	(813.0)	1,121	165	(579.4)	575	572	(0.5)
Missouri	25,868	25,698	(0.7)	3	3	(0.0)	1,015	975	(4.1)	656	667	(−1.7)
Nebraska	5,443	5,262	(3.4)	5	20	(−75.0)	141	141	(0.0)	341	337	(1.2)
North Dakota	1,957	1,769	(10.6)	15	—		30	29	(3.5)	103	73	(41.1)
South Dakota	3,015	2,145	(40.6)	1	1	(0.0)	58	30	(93.3)	198	156	(26.9)
**South Atlantic**	249,979	194,409	(28.6)	4,466	3,778	(18.2)	1,632	1,551	(5.2)	14,478	13,488	(7.3)
Delaware	4,718	4,718	(0.0)	984	952	(3.4)	13	13	(0.0)	142	137	(3.7)
District of Columbia	6,549	6,414	(2.1)	61	20	(205.0)	7	3	(133.3)	100	26	(284.6)
Florida	72,931	71,731	(1.7)	110	127	(−13.4)	497	500	(−0.6)	6,741	6,749	(−0.1)
Georgia	39,828	29,934	(33.1)	40	53	(−24.5)	223	194	(15.0)	2,362	2,365	(−0.1)
Maryland	23,747	22,138	(7.3)	2,024	1,775	(14,0)	148	134	(10.5)	803	784	(2.4)
North Carolina	41,045	—		96	63	(52.4)	220	223	(−1.4)	1,810	1,053	(71.9)
South Carolina	26,654	25,014	(6.7)	42	39	(7.7)	262	252	(4.0)	1,195	1,153	(3.6)
Virginia	30,903	30,881	(0.1)	908	579	(56.8)	222	198	(12.1)	1,095	1,004	(9.1)
West Virginia	3,604	3,579	(0.7)	201	170	(18.2)	40	34	(17.7)	230	217	(6.0)
**Eastern South Central**	92,522	87,926	(5.2)	41	36	(13.9)	803	760	(5.7)	3,077	2,937	(4.8)
Alabama	25,929	22,833	(13.6)	3	3	(0.0)	305	285	(7.0)	932	850	(9.7)
Kentucky	13,293	13,166	(1.0)	1	1	(0.0)	226	219	(3.2)	453	451	(0.4)
Mississippi	23,589	22,146	(6.5)	—	—		75	66	(13.6)	899	853	(5.4)
Tennessee	29,711	29,781	(−0.2)	37	32	(15.6)	197	190	(3.7)	793	783	(1.3)
**Western South Central**	162,915	136,836	(19.1)	278	48	(479.2)	3,993	2,882	(38.6)	6,411	4,751	(34.9)
Arkansas	14,354	—		—	—		369	278	(32.7)	615	607	(1.3)
Louisiana	27,628	25,308	(9.2)	—	—		149	90	(65.6)	1,180	599	(97.0)
Oklahoma	15,023	12,959	(15.9)	2	—		117	77	(52.0)	652	615	(6.0)
Texas	105,910	98,569	(7.5)	276	48	(475.0)	3,358	2,437	(37.8)	3,964	2,930	(35.3)
**Mountain**	80,476	73,912	(8.9)	57	44	(30.0)	1,019	890	(14.5)	3,028	2,812	(7.7)
Arizona	26,002	25,110	(3.6)	7	6	(16.7)	277	224	(23.7)	1,086	1,051	(3.3)
Colorado	19,998	16,362	(22.2)	1	1	(0.0)	231	233	(−0.9)	619	621	(−0.3)
Idaho	3,842	3,501	(9.7)	16	15	(6.7)	99	99	(0.0)	174	172	(1.2)
Montana	2,988	2,913	(2.6)	3	3	(0.0)	61	57	(7.0)	110	99	(11.1)
Nevada	10,045	9,743	(3.1)	13	5	(160.0)	24	9	(166.7)	252	173	(45.7)
New Mexico	9,493	8,947	(6.1)	5	5	(0.0)	85	66	(28.8)	369	325	(13.5)
Utah	6,145	5,466	(12.4)	9	7	(28.6)	220	181	(21.6)	321	283	(13.4)
Wyoming	1,963	1,870	(5.0)	3	2	(50.0)	22	21	(4.8)	97	88	(10.2)
**Pacific**	190,872	179,087	(6.6)	178	207	(−14.0)	1,517	1,062	(42.8)	6,662	6,300	(5.8)
Alaska	5,166	4,412	(17.1)	7	3	(133.0)	59	49	(20.4)	68	70	(−2.9)
California	146,796	139,689	(5.1)	117	154	(−24.0)	869	473	(83.7)	5,003	4,757	(5.2)
Hawaii	6,026	5,610	(7.4)	—*	—*		46	29	(58.6)	338	297	(13.8)
Oregon	11,497	10,245	(12.2)	38	35	(8.6)	252	246	(2.4)	433	416	(4.1)
Washington	21,387	19,131	(11.8)	16	15	(6.7)	291	265	(9.8)	820	760	(7.9)

* Not notifiable in Hawaii.

## References

[1] Smallman-Raynor M, Cliff AD, Haggett P, Stroup DF, Williamson GD (1999). Spatial and temporal patterns in final amendments to provisional disease counts. J Public Health Manag Pract.

[2] CDC (2011). Summary of notifiable diseases—United States, 2009. MMWR.

[3] CDC (2006). Notice to readers: changes in presentation of data from the National Notifiable Diseases Surveillance System. MMWR.

[4] Stroup DF, Williamson GD, Herndon JL, Karon JM (1989). Detection of aberrations in the occurrence of notifiable diseases surveillance data. Stat Med.

[5] Stroup DF, Wharton M, Kafadar K, Dean AG (1993). Evaluation of a method for detecting aberrations in public health surveillance system data. Am J Epidemiol.

[6] Birkhead G, Chorba TL, Root S, Klaucke DN, Gibbs NJ (1991). Timeliness of national reporting of communicable diseases: the experience of the National Electronic Telecommunications System for Surveillance. Am J Public Health.

[7] Boehmer TK, Patnaik JL, Burnite SJ, Ghosh TS, Gershman K, Vogt RL (2011). Use of hospital discharge data to evaluate notifiable disease reporting to Colorado’s Electronic Disease Reporting System. Public Health Rep.

[8] Silk BJ, Berkelman RL (2005). A review of strategies for enhancing the completeness of notifiable disease reporting. J Public Health Manag Pract.

